# Improved disinfection performance for 280 nm LEDs over 254 nm low-pressure UV lamps in community wastewater

**DOI:** 10.1038/s41598-023-34633-7

**Published:** 2023-05-10

**Authors:** Sean A. MacIsaac, Kyle D. Rauch, Taylor Prest, Richard M. Simons, Graham A. Gagnon, Amina K. Stoddart

**Affiliations:** 1 Centre for Water Resources Studies, Dalhousie University Halifax, Halifax, NS B3H 4R2 Canada; 2 AquiSense Technologies, Erlanger, KY 41018 USA

**Keywords:** Engineering, Civil engineering

## Abstract

Ultraviolet (UV) disinfection has been incorporated into both drinking water and wastewater treatment processes for several decades; however, it comes with negative environmental consequences such as high energy demands and the use of mercury. Understanding how to scale and build climate responsive technologies is key in fulfilling the intersection of UN Sustainable Development Goals 6 and 13. One technology that addresses the drawbacks of conventional wastewater UV disinfection systems, while providing a climate responsive solution, is UV light emitting diodes (LEDs). The objective of this study was to compare performance of bench-scale 280 nm UV LEDs to bench-scale low pressure (LP) lamps and full-scale UV treated wastewater samples. Results from the study demonstrated that the UV LED system provides a robust treatment that outperformed LP systems at the bench-scale. A comparison of relative energy consumptions of the UV LED system at 20 mJ cm^−2^ and LP system at 30 and 40 mJ cm^−2^ was completed. Based on current projections for wall plug efficiencies (WPE) of UV LED it is expected that the energy consumption of LED reactors will be on par or lower compared to the LP systems by 2025. This study determined that, at a WPE of 20%, the equivalent UV LED system would lead to a 24.6% and 43.4% reduction in power consumption for the 30 and 40 mJ cm^−2^ scenarios, respectively.

## Introduction

Ultraviolet (UV) disinfection has been incorporated into both drinking water and wastewater treatment processes for several decades. Conventional UV disinfection is driven by mercury-halogen lamps which emit germicidal light at 254 nm. While effective in inactivation of a wide array of pathogens across a variety of water matrices, mercury-based UV disinfection poses an environmental concern as the mercury used for light generation in the lamps is toxic, the lamps operate at maximum energy efficiency between 30 and 35% creating a high energy demand^1^, and the high operating temperatures of the lamps cause issues of organic and inorganic fouling of the protective quartz lamp sleeves which decreases the efficacy of UV disinfection^2^.

UN Sustainable Development Goal (SDG) 6 focuses on the sanitation and cleanliness of water^3^. Jarvis has noted that sustainable control over microorganisms is key in achieving this goal^4^. Novel technologies that are robust enough to address the many issues involved with achieving SDGs must be studied^5^. Understanding how to scale and build climate responsive technologies is key in fulfilling the intersection of SDG6 and SDG13, Climate Action, in a timely manner. UV light emitting diodes (LEDs) address the previously mentioned drawbacks of conventional wastewater UV disinfection, while providing a climate responsive solution^6–9^.

UV LEDs function similarly to conventional mercury-halogen lamps but have a formfactor akin to a typical visible light LED and do not use mercury for the generation of UV photons. UV LEDs have matured as a technology over the course of the last decade to the point where full-scale use is imminent and commercial point-of-use systems are readily available^8,10,11^. One of the opportunities provided by UV LED technology is increased germicidal efficiency from the emittance of different wavelengths of UVC light. Subtle shifts in UV wavelength can substantially improve disinfection performance^12–14^. This relative shift in germicidal efficacy is unique to each microorganism and is known as an action spectra, and is related to the relative abundance of the nucleotide base pairs in the organism’s DNA^15^. The increase in germicidal efficiency from shifting the wavelength can decrease the required fluence need to achieve a similar log reduction to help offset the lower energy efficiency currently experienced by UV LEDs in the UVC range.

UV LEDs are modular and can be scaled to the application, meaning that they have been used in applications ranging from remote point-of-use disinfection to pilot-scale systems^9,16^. Tuning of the emitted light by using an array of UV LED chips broadens the design space and range of applications for UV disinfection^6^. UV LED reactors have been considered with a bespoke array of LEDs that are tailored to a specific influent wastewater matrix and UV absorbance properties^17,18^. While UV LEDs offer many benefits, in their current state there are some characteristics that may hinder larger scale implementation. This includes shorter lamp lifespans, although high quality UVC LEDs can already achieve lifespans of 10,000 h similar to low-pressure mercury lamps^9,19^. Additionally, UV LEDs have an increased capital cost per watt of optical output compared to low-pressure mercury lamps ($100–400/W compared to $2/W); however, this difference has shrunk substantially over the last decade with the trend expected to continue as UV LEDs mature as a technology^19^. To date, there has been no full-scale implementation of a UV LED reactor in a wastewater treatment facility.

Choosing the right tool for assessing the performance of UV technologies is also key in scaling emergent technologies. Several assessment techniques exist (e.g., collimated beam testing, fluorescent microspheres, CFD modelling, and biodosimetry), but they are often limited in either long term assessment capability, representative full-scale results, or both. UV audits have been identified as an approach that identifies the full-scale performance of wastewater treatment facilities (WWTFs) while simultaneously comparing that performance to independent light sources^20^.

Another consideration for UV LEDs is the wavelength dependent interactions with UV absorbing and blocking contaminants in a wastewater matrix. The complex nature of wastewater matrices often limits UV disinfection. Typically, wastewater matrices have lower UV transmittance (UVT%) as the UV wavelength decreases limiting the penetration of UV light at typical UV disinfection wavelengths of 254 nm. As such, there is potential to use longer UVC wavelengths with better penetration capabilities and similar germicidal efficiencies to improve disinfection performance in a wastewater treatment facility; however, exploration of treatment efficacy using alternate UV LED wavelengths in wastewater has largely been left unexplored.

The objective of this study was to compare bench-scale 280 nm UV LEDs to bench-scale low pressure (LP) lamps and full-scale LP UV treated wastewater samples through an SDG lens. The use of UV auditing provides a tool for comparing the potential efficacy of a full-scale UV LED system operating at an equivalent fluence to current LP based full-scale systems. This work examined fluences ranging from 10 to 40 mJ cm^−2^ for two (UV LED and LP) collimated beam units while simultaneously collecting UV treated samples from a full-scale system. Additionally, as UV LED wall plug efficiency is rapidly improving and there is a knowledge gap regarding the state of the art for UV LEDs, the current and projected energy savings from using UV LEDs compared to conventional UV systems was calculated and implications for carbon reductions from a Canadian perspective were examined.

## Methods

### Wastewater treatment facility site description

The monitored wastewater treatment facility (44° 48′ 52.4016ʺ N, 63° 43′ 55.308ʺ W) uses a secondary activate sludge system followed by a closed channel UV disinfection system with a maximum design flow of 1363 m^3^ day^−1^ and a design fluence of 30 mJ cm^−2^. The facility provides wastewater for a service population of approximately 930. Wastewater was collected weekly from the wastewater treatment facility for the first four weeks of the study and collected twice per week for the remaining eight weeks of the study. Plant UV-treated samples were also collected along with the untreated samples to compare the performance of the full-scale facility to bench-scale disinfection using LP and UV LED collimated beam units. Wastewater samples were transported to the lab on ice and analyzed the day of collection.

### Bench-scale apparatus description

A Calgon Carbon collimated beam unit was used for all LP bench-scale work. The LP lamp was turned on 30 min to allow for the lamp to warm up and ensure the system was operating at full power prior to measuring the irradiance. An AquiSense Pearlbeam UV LED collimated beam unit with a nominal 280 nm UV LED emitting at peak wavelength of 279 nm was used for all LED bench-scale work. All UV irradiance was measured using an OceanOptics USB2000 spectroradiometer and the appropriate correction factors described by Bolton and Linden were applied to the measured irradiance prior to calculating exposure time for target fluences^21^. Fluences of 10, 20, 30, and 40 mJ cm^−2^ were used for both the LP and UV LED samples. All bench-scale samples and full-scale pre- and post-UV samples were enumerated for *E. coli* following the enumeration protocols below.

### Water quality

Total suspended solids (TSS) and total iron were collected to assess the impact of water quality on disinfection performance. TSS was conducted as per *The Standard Methods for Examination of Water and Wastewater*^22^. Total iron was measured following the USEPA FerroVer Method (Method 8008) on a DR5000 spectrometer. UVT% was collected at 254 and 279 nm using a 1 cm quartz cuvette on a DR5000 spectrometer. Flow rate and UV intensity were collected from the control panel of the full-scale UV system at the time of sampling.

### Treatment protocol and *E. coli* enumeration

52 mL of untreated wastewater was added to a sterile petri dish and gently mixed using a stir bar. Wastewater samples were then exposed to UV light for each of the required fluences and UV light sources while operating under subdued red light to minimize the effect of photo-repair. Samples were then immediately transferred to a sterile Colilert bottle and diluted with phosphate buffer solution. A Colilert packet was then added to each bottle and mixed before transferring the solution to Quantitrays and then incubated for 24 h at 37 °C. Samples were then counted and quantified for *E. coli*. Well counts were converted to MPN-100 mL^−1^ using the quantitray package in R.

### Statistical methods and data visualization

Geeraerd’s non-linear heat inactivation model was adapted to capture kinetics with a log linear and shoulder phase as in Eq. (1) ^23^.

1 $${N}_{t }= ({N}_{0}-{N}_{res}){10}^{-kD}+{N}_{res}$$

All statistics, models and figures were developed using the R (V 4.0.3)^24^ using core R functions and the following additional packages: nls, nlstools, and ggplot, Additionally, Affinity Designer (V 1.10.5) was used for all other illustrations and graphics that were developed for this study^25^.

### Energy considerations

The fluence rate within a reactor is a function of several factors relating to its design and operation as well as the absorbance of the water being treated; considering all reactor characteristics to be constant between the full-scale and an equivalent LED system the relationship can be condensed to Eq. (2), where *H'*_*e*_ is the mean fluence rate, *q* is the UV power into the system, and α is the absorption coefficient of the water.

2 $${H}^{\prime}_{e}\propto \frac{q}{\alpha }$$

Relative power consumptions were then calculated by dividing the mean fluence rate by the current typical wall-plug efficiencies (7.1% WPE).

## Results and discussion

### Water quality

The influent wastewater quality over the duration of the sampling period is shown in Table 1. UVT_254_ ranged from 39.7 to 70.6% (mean = 61.8%) and UVT_279_ ranged from 44.7 to 75.7% (mean = 66.8%) and flow ranged from 164 to 1010 m^3^ day^−1^ (mean = 490 m^3^ day^−1^). These data indicate that the water quality and flow was variable over the sampling period and further strengthens the disinfection data because a variety of wastewater conditions were captured. The TSS was observed to be relatively low for a wastewater facility with a mean value of 5.5 mg L^−1^ and a max value of 9.5 mg L^−1^. Other facilities in the region have previously reported mean TSS concentrations between 9.7 and 23.9 mg L^−1^^20^. Mean total iron concentrations were observed to be 0.21 mg L^−1^ with a max value of 0.36 mg L^−1^. which are below or near the value (0.3 mg L^−1^) expected to impact disinfection performance^26^. Based on previous work, the TSS and iron concentrations measured in this study suggest that the matrix should respond well to UV treatment.

**Table 1:** Mean, minimum, and maximum values for select water quality parameter from duration of study.

Parameter	Mean (95% CI)	Min	Max	n
Flow, m^3^ day^−1^	490 (361–618)	164	1009	15
Full-Scale Intensity, mW cm^-2^	27 (19.2–34.8)	11.1	55.1	15
Influent *E. coli*, MPN-100 mL^−1^	58,044 (1896–114,191)	7270	303,679	12
Total Iron, mg L^−1^	0.21 (0.16–0.25)	0.09	0.36	16
TSS, mg L^−1^	5.5 (4.2–6.9)	1.0	9.5	16
UVT_254_, %	61.8 (58–65.6)	39.7	70.6	16
UVT_279,_ %	66.8 (63–70.6)	44.7	75.7	16

Full-scale plant data were also collected as part of the typical sampling for the duration of the study period. Operators collected flow, influent and effluent TSS, influent and effluent pH and effluent *E. coli* concentrations approximately every 2 weeks. Table 2 summarizes the parameters relevant to this study. Comparing the full-scale data to the bench-scale data indicates that the range of flows and wastewater quality captured during the sampling period captured the range of typical flows for the full-scale facility. Average flows at the plant over the duration of the study were 471 m^3^ day^−1^ (average for sampling events = 490 m^3^ day^−1^). The average TSS at the facility was 6.8 mg L^−1^ which was a slightly higher than the 5.5 mg L^−1^ observed in the lab.

**Table 2:** Full-scale quality data during experiment sampling period collected by operators as part of their regular sampling procedures.

Sample	Daily Flow, m^3^ day^−1^	Effluent TSS, mg L^−1^	Effluent *E. coli*, CFU-100 mL^−1^
1	632	8.6	70
2	638	11.6	< 10
3	379	4.4	< 10
4	312	10.2	< 10
5	415	1.0	< 10
6	446	5.0	30

### Treatment performance

**Figure 1:**
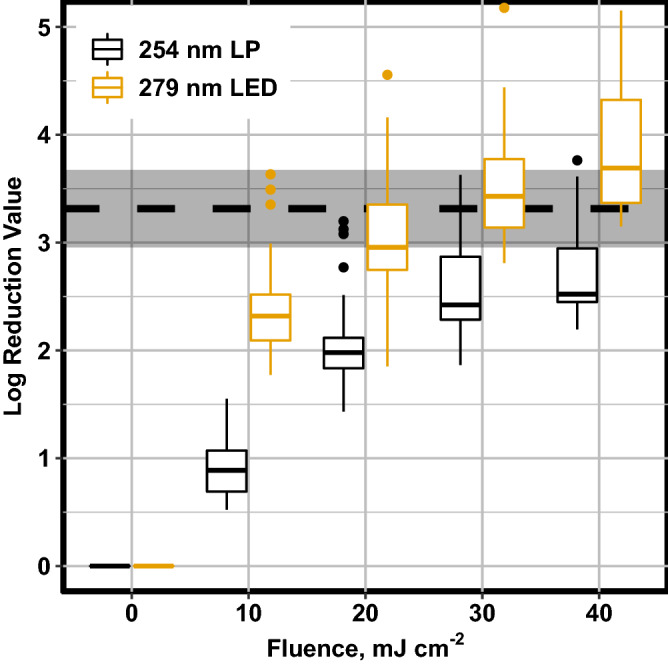
Log reduction values for UV LED and LP treated wastewater. The dashed line and shaded region represent the mean full-scale plant performance and 95% confidence interval over the duration of sampling (n = 12). The centerline of the boxplot represents the median value, while the upper and lower hinge represent the 1st and 3rd quartile, respectively. The whiskers represent 1.5*the interquartile range, and outlying data points are plotted as points.

Figure 1 shows the disinfection performance for the wastewater treatment facility (WWTF) for both LED and LP collimated beam light sources. The plant performance is shown by the grey bar and dashed line for comparison to the bench-scale treatments indicated by the different colored box plots. The design fluence for the reactor installed at the WWTF was 30 mJ cm^−2^, and these results show that UV LEDs at 279 nm outperform the LP at this fluence. Furthermore, the overlap of the plant performance shaded region and the LP bench-scale treatment at the 30 and 40 mJ cm^−2^ in Fig. 1 indicate that the system was matrix limited when considering the UV auditing methodology, (i.e. the system is treating the wastewater at the highest quality)^20^. This was not unexpected as the average daily flows experienced by the facility are only 36% of the design flows. The LED light source outperformed the LP collimated beam at each of the examined fluences at the bench-scale. This result suggests that UV LED light sources are a better tool for disinfection under some wastewater quality conditions.

Collimated beam results for the LP light source indicated that it only achieved disinfection comparable to full-scale LP performance at a fluence greater than 40 mJ cm^−2^. The design fluence of the WWTF is 30 mJ cm^−2^ which suggests that the facility is performing above the design rate. This is not surprising as the average flowrate experienced at the facility was 490 m^3^ day^−1^ over the course of the study compared to the design flow of 1363 m^3^ day^−1^. These data show that a significant amount of energy is wasted by the LP system due to an excessive applied UV fluence. A full-scale LED system installed at this WWTF could be better tuned to the changing water quality at this location.

### Inactivation kinetics modelling

**Figure 2:**
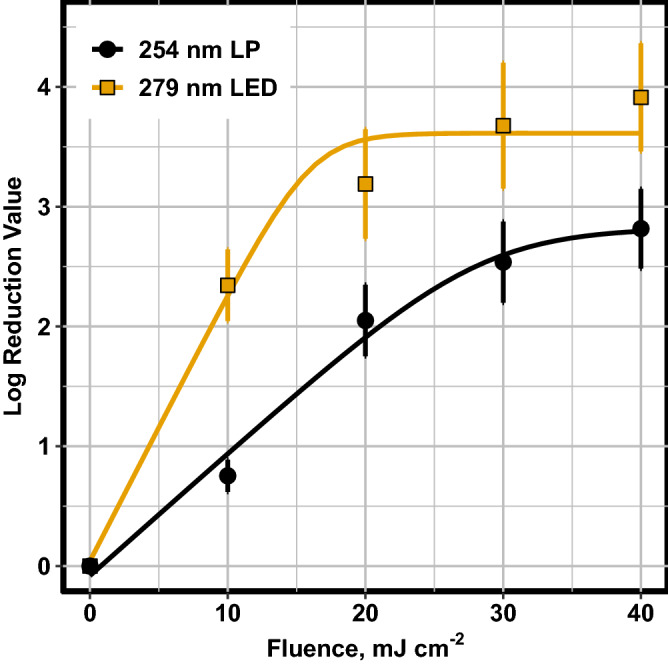
Geeraerd’s model comparison between 254 nm LP and 279 nm UV LED. Error bars represent 95% confidence interval on the mean (n = 12) as indicated by black (LP) and yellow (LED) points.

Modelling of each of the disinfection light sources further indicated that there were significant differences in the behaviour of disinfection between the LP and LED treatments The kinetics for the LED light source was observed to shift from the log-linear to the shoulder phase near a fluence of 20 mJ cm^−2^ (Fig. 2). The LP modelling indicated that the shouldering phase begins at 40 mJ cm^−2^ and would reach steady state at a fluence that was beyond the range of fluences examined in this study.

Table 3 shows the kinetics data and Geeraerd’s model fit for each of the light sources and WWTF data. The effectiveness of the LED versus LP was observed to be significantly different. The 279 nm LED was found to have a k-value that was twice that of the LP system. Practically this means that the 279 nm UV LED requires half the fluence to achieve the same log reduction in *E. coli.* The increased k-value could be attributed to difference in germicidal efficacy of the two wavelengths examined or to other inactivation mechanisms related to protein damage; however, a study by Beck et al.^17^ found that there was no increased synergy when using combined wavelength treatment with 280 nm UV LEDs. The authors suggest that the lack of synergy indicates that the main mechanism for inactivation at 280 nm is through DNA absorption and pyrimidine dimer formation. Thus, the difference in kinetics found in this study may be related to increased germicidal efficacy at 279 nm.

**Table 3:** Kinetics data for Geeraerd’s model fit parameter and Plan LRV for 2 UV Lamps (bracketed values indicate a 95% confidence interval about the mean, n = 12).

Lamp	k (cm^2^ mJ^−1^)	N_res_ Log (MPN-100 mL^−1^)	Plant LRV Log (MPN-100 mL^−1^)	RMSE
254 nm LP	0.102 (0.085–0.119)	2.82 (2.58–3.07)	3.31 (2.76–3.87)	0.405
279 nm LED	0.223 (0.173–0.274)	3.61 (3.39–3.84)		0.598

The 279 nm UV LED N_res_, or the upper level of treatment, was significantly greater compared to the LP system (3.61 log versus 2.82 log). As this upper limit of disinfection is typically due to particle shielding effects, this suggests that the 279 nm UV LED had a higher propensity to reach bacterial communities that may have attached to the particulate matter in the matrix. Particle shielding effects have been observed to be wavelength dependent, as particle UV absorbance capability increases as the wavelength decreases, which lowers the inactivation capabilities at those lower wavelengths^27^. Furthermore, self aggregation of *E. coli* has also been shown to be wavelength dependent^28,29^.

The confidence intervals for each bench-scale light source N_res_ values and WWTF performance overlapped and there was no significant difference between the bench-scale treatments and full-scale disinfection performance. This is the first instance where the auditing process captured a plant that was substantially overdosing UV radiation. This result indicates that the UV auditing process improves operational efficiency even for a plant which is operating under ideal disinfection outcomes.

### Energy implications

The increase in the upper level of treatment (+ 0.79 log) observed for the UV LED source suggests that the interaction of the wavelengths and particulate matter may be influencing performance. Nonetheless, the 33% improved germicidal efficiency when compared to traditional UV light sources begins to address the current discrepancies in wall plug efficiency (WPE) between the two technologies. As of 2020, the highest WPE achieved for a commercially available 280 nm UV LED was 4.1% (LP lamps 30–35%) and external quantum efficiency (EQE) was 6.1%^30^. Currently, UV LEDs in the 280 nm ± 5 nm range have an EQE ranging from 9 to 20.3%^7,31^ and best LEDs would typically be around 7.1% WPE. This marked improvement in the last few years, and forecasted improvements for UV LED WPE indicate that the energy efficiency discrepancy will decrease as LED light sources improve^32,33^. Further efficiency can be found through creative design of UV LED reactors such as highly reflective internal surfaces and overall shape of the reactor which allows for maximum interaction of emitted light and target pathogen. These efficiencies combined can improve the feasibility of full-scale implementation of UV LED reactors.

**Figure 3:**
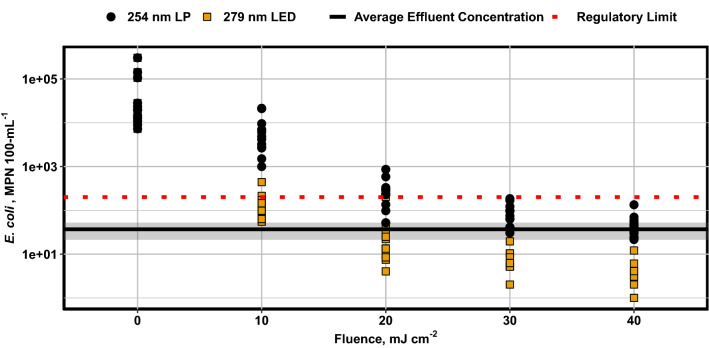
*E. coli* concentration versus fluence for two UV systems. Black circles represent bench-scale treatment at 254 nm using a LP collimated beam system. Yellow squares represent bench-scale treatment at 279 nm using an UV LED collimated beam system. The red dotted line represents target effluent concentration to meet regulatory discharge requirements. The black line and shaded region represent the mean and 95% confidence interval (n = 12) of the effluent concentration from post UV samples.

UV LEDs achieved similar disinfection performance to the full-scale WWTF at a UV LED fluence of 20 mJ cm^−2^ whereas the full-scale design fluence was 30 mJ cm^−2^ (Fig. 3). It has been illustrated above that the full-scale LP installation was operating to deliver a fluence more than 40 mJ cm^−2^ reduction equivalent fluence (REF), despite a design fluence of 30 mJ cm^−2^, and hence is consuming additional power to treat above the required level. It was also shown that an equivalent level of inactivation could be achieved by an LED system operating to deliver a 20 mJ cm^−2^ REF. A detailed analysis of the energy cost comparison of an equivalent LED system installed at the Springfield Lake site is beyond the scope of this paper, though a baseline comparison may be drawn. Therefore, the fluence to be delivered by an equivalent LED system would be 1.5–2.0 times lower than that of the current LP installation.

UVT data collected during the study showed that the absorption coefficient of the water was 16% lower at 279 nm than at 254 nm, resulting in a 19.3% higher fluence per unit UV power. Combining these factors gives a UV power demand of the equivalent LED system of 42–56% of the LP installation. A cutting-edge commercial UV-C LED would achieve approximately 7.5% electrical efficiency and would lose approximately 5% in power conversion and distribution, giving an approximate WPE of 7.1%. The UV lamps of LP systems typically see 30% WPE^15^. Applying these electrical efficiency factors, the equivalent LED system would consume 59–113% more power than the LP installation in continuous operation.

For the UV LED system to be comparable in energy consumption to the 30 mJ cm^−2^ scenarios, the WPE of the system would need to reach 15.1%, and for the 40 mJ cm^−2^ scenario the WPE would only need to be 11.3%. Based on projections for UV LEDs, the WPE of UV LEDs between 265 and 280 nm is expected to reach approximately 20% by 2025^34^. At a WPE of 20%, the equivalent UV LED system would lead to a 24.6% and 43.4% reduction in power consumption for the 30 and 40 mJ cm^−2^ scenarios, respectively. Examining these scenarios indicates that in the future, UV LED reactors will have the potential to match or even outperform LP systems in terms of energy consumption by taking advantage of the difference in germicidal efficiency of alternate wavelengths even though the WPE of the lamps have not reached parity.

The enhanced performance when comparing the gross energy usage for LED and LP light sources is further enhanced by the practical benefits of full-scale day-to-day use of LED technologies. For example, UV LEDs can be dimmed, brightened, and shut off during times when disinfection is not required. LP systems are typically only ever shut off during routine maintenance or system repairs meaning that energy usage is not optimized throughout the lifetime of UV lamps. Additional research and assessment of these inherent improvements is needed to better quantify the benefits of UV LEDs over traditional disinfection technologies.

### Sustainability implications

As of 2020, there were 1866 wastewater treatment plants in Canada. Approximately 25% of the WWTF, minus lagoons, were commissioned between 2010 and 2020^35^. Canadian WWTFs are on average 17.3 years old and approximately 48.9% through the useful lifespan^36^. These data indicate that in the next 18 years large upgrades to sewage treatment facilities in the country will be needed. UV LED technologies are projected to further mature by this time and upgrades for existing wastewater treatment facility UV systems would contribute to a more sustainable future.

**Figure 4:**
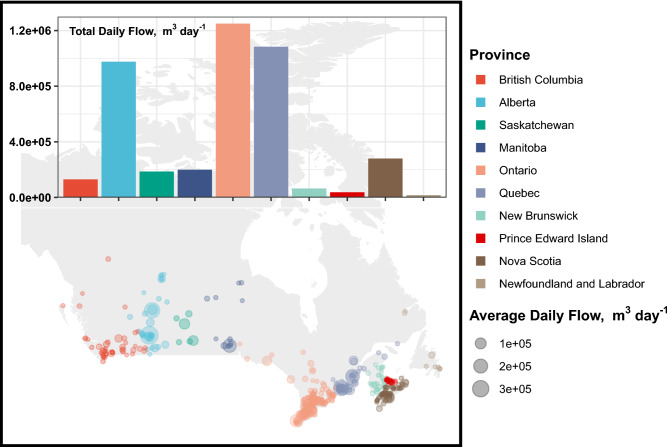
Location and size of wastewater treatment facilities in the 10 Provinces of Canada that use UV for disinfection. Insert represent total daily flows of WWTFs using UV treatment per Province in Canada.

The Wastewater Systems Effluent Regulation reporting documentation collects effluent and system data from wastewater treatment facilities across Canadian Provinces with average daily flows greater than 100 m^3^ and located below the 54th parallel^37^. This dataset includes information on 601 wastewater treatment facilities of these is estimated that 421 use UV disinfection as the final treatment prior to discharge. Location and size of these systems based on average daily flow is provided in Fig. 4. Ontario, followed by Quebec and Alberta are the top three users of UV disinfection systems in Canada.

Using flowrate as a measure of how large a UV system must be to deliver a 30 mJ cm^−2^ fluence, an estimation of current energy consumption for UV treatment systems was completed. Using the Springfield Lake treatment facility with a baseline flow of 1363 m^3^ day^−1^ and an annual energy usage of 5781.6 kWh, estimations of annual power consumption across the provincial totals were calculated. Further, using the provincial energy profiles provided by the Government of Canada, the annual CO_2(e)_ generated was estimated^38^. These values were then used to assess the scenario where all facilities switch to 20% WPE efficient UV LED systems. This analysis indicates that an annual reduction of 946 tonnes of CO_2(e)_ is attainable by operating UV LED disinfection systems (Table 4).

**Table 4:** Estimated CO_2(e)_ generation from wastewater treatment facilities using UV treatment based on 2022 provincial rates of CO_2(e)_ generated/kWh and total daily flows.

Province	CO_2(e)_kW h^−1^	Total daily average flow, m^3^ day^−1^	Number of facilities	Power, kWh year^−1^	Annual CO_2(e)_, Tonnes year^−1^	Estimated annual CO_2(e)_ for LEDs in 2025, Tonnes year^−1^
BC	7.3	129,784	45	550,521	4.02	3.03
AB	590	975,988	33	4,139,965	2443	1842
SK	580	186,428	6	790,794	459	346
MB	1.1	199,519	12	846,324	0.931	0.702
ON	25	1,250,231	172	5,303,253	133	100
QC	1.5	1,084,258	39	4,599,226	6.90	5.20
NB	290	64,587.8	28	273,969.8	79.5	59.9
NL	24	16,225.2	8	68,824.37	1.65	1.25
NS	670	280,726.5	59	1,186,549	795	599
PE	0	36,575	19	155,144.5	0	0

Energy consumption alone is not a full measure of the sustainability of a treatment system, and other aspects captured in a Life Cycle Assessment (LCA) such as material generation, use, lifespan, and disposal can contribute to the overall carbon footprint of a treatment technology. A recent study by McKee and Chatzisymeon^39^ used a LCA to examine the sustainability differences between a UV LED/TiO_2_ and a mercury-based UV/TiO_2_ photocatalytic treatment system to remove bisphenol-A from polluted water at the bench-scale. The authors found that the UV LED treatment reduced the environmental impact by 40% and suggest most of the reduction is due to the reduced energy consumption, increased lifespan, and the mercury-free nature of the UV LED unit. Furthermore, the authors note that the majority of the environmental impact is related to the energy consumption of treatment, indicating that reducing the energy consumption will have the largest impact on the sustainability, While a full LCA is beyond the scope of this current study, the work from McKee and Chatzisymeon suggest that, as the difference in energy efficiencies between UV LEDs and mercury-based systems shrinks, paired with the treatment efficiency gained from using targeted UV LED wavelengths, the overall sustainability of UV LED treatment will continue to improve compared to mercury-based systems.

## Conclusion

The outcomes of this study indicate that UV LED technologies are capable of sufficient full-scale performance and can outperform traditional technologies at congruent fluences. The changes in water quality at the WWTF over the course of sampling also indicate that UV LED disinfection provides robust treatment. Additionally, the energy comparisons completed for this study indicate that UV LED systems have the potential to provide a similar performance at a lower power consumption in the very near future. Improvements in disinfection efficacy at comparable fluences have greater implications as UV LED technologies are brought to full-scale devices. As infrastructure is replaced to maintain the safety of drinking water and wastewater treatment processes, UV LEDs provide a scalable tool to respond to the pressures of climate change. Future work is recommended using this approach to compare LED performance to traditional technologies. using a truly full-scale reactor that is installed into the network of a municipal wastewater treatment facility. UV LED systems should also be better quantified to understand the potential energy savings gained from other unique features such as instantaneous dimming and brightening of the LEDs.

## Data Availability

The datasets generated and analyzed during the current study are available from the corresponding author on reasonable request.
